# Highly Pathogenic Avian Influenza Virus Subtype H5N1 in Africa: A Comprehensive Phylogenetic Analysis and Molecular Characterization of Isolates

**DOI:** 10.1371/journal.pone.0004842

**Published:** 2009-03-17

**Authors:** Giovanni Cattoli, Isabella Monne, Alice Fusaro, Tony M. Joannis, Lami H. Lombin, Mona M. Aly, Abdel S. Arafa, Katharine M. Sturm-Ramirez, Emmanuel Couacy-Hymann, Joseph A. Awuni, Komla B. Batawui, Kodzo A. Awoume, Gilbert L. Aplogan, Adama Sow, Andrè C. Ngangnou, Iman M. El Nasri Hamza, Djibo Gamatié, Gwenaelle Dauphin, Joseph M. Domenech, Ilaria Capua

**Affiliations:** 1 OIE/FAO Reference Laboratory for avian influenza and Newcastle disease, Istituto Zooprofilattico Sper.le delle Venezie, Legnaro (PD), Italy; 2 National Veterinary Research Institute, Vom, Nigeria; 3 National Laboratory for quality control on poultry production, Dokki (Cairo), Egypt; 4 Fogarty International Center, National Institutes of Health, Bethesda, Maryland, United States of America; 5 Laboratoire Central de Pathologie Animale, Bingerville, Ivory Coast; 6 Accra Veterinary Laboratory, Accra, Ghana; 7 Laboratoire Central Vétérinaire, Lomé, Togo; 8 Laboratoire de Diagnostic Vétérinaire et de Sérosurveillance, Parakou, Bénin; 9 Laboratoire National d'Elevage, Ouagadougou, Burkina Faso; 10 Laboratoire National Vétérinaire, Garoua, Cameroon; 11 Central Veterinary Research Laboratories, Khartoum, Sudan; 12 Direction des Laboratoires Vétérinaires, Niamey, Niger; 13 Food and Agriculture Organization of the United Nations, AGAH Division, Rome, Italy; University of Maryland, United States of America

## Abstract

Highly pathogenic avian influenza virus A/H5N1 was first officially reported in Africa in early 2006. Since the first outbreak in Nigeria, this virus spread rapidly to other African countries. From its emergence to early 2008, 11 African countries experienced A/H5N1 outbreaks in poultry and human cases were also reported in three of these countries. At present, little is known of the epidemiology and molecular evolution of A/H5N1 viruses in Africa. We have generated 494 full gene sequences from 67 African isolates and applied molecular analysis tools to a total of 1,152 A/H5N1 sequences obtained from viruses isolated in Africa, Europe and the Middle East between 2006 and early 2008. Detailed phylogenetic analyses of the 8 gene viral segments confirmed that 3 distinct sublineages were introduced, which have persisted and spread across the continent over this 2-year period. Additionally, our molecular epidemiological studies highlighted the association between genetic clustering and area of origin in a majority of cases. Molecular signatures unique to strains isolated in selected areas also gave us a clearer picture of the spread of A/H5N1 viruses across the continent. Mutations described as typical of human influenza viruses in the genes coding for internal proteins or associated with host adaptation and increased resistance to antiviral drugs have also been detected in the genes coding for transmembrane proteins. These findings raise concern for the possible human health risk presented by viruses with these genetic properties and highlight the need for increased efforts to monitor the evolution of A/H5N1 viruses across the African continent. They further stress how imperative it is to implement sustainable control strategies to improve animal and public health at a global level.

## Introduction

Since the earliest known progenitor virus detected in China, A/goose/Guandong/96, numerous genetic lineages of highly pathogenic avian influenza (HPAI) viruses of H5N1 subtype (indicated as A/H5N1 from now on) have emerged and spread. In 2005 a large scale outbreak of A/H5N1 led to the death of thousands of migratory waterfowl in Qinghai Lake in North-West China. Subsequently, the A/H5N1 virus spread westward, from Qinghai Lake through Central Asia, Europe, the Middle East and Africa [1]. The spread of A/H5N1 viruses across Africa raises serious concerns regarding the sustainability of the poultry sector and public health issues. The latter include both food security aspects for low-income countries and the potential threat to human health due to the extensive circulation of avian influenza viruses capable of causing significant mortality in humans.

After its first emergence in poultry farms in early 2006 in Nigeria [2], A/H5N1 virus was detected in many other African countries. The first outbreak was recorded in Kaduna State, Nigeria, in mid-January 2006 and in less than a month, the virus was identified in Egypt, Niger and Cameroon. In April 2006 the virus was also detected in Sudan, Burkina Faso, Djibouti and Ivory Coast. A year later the virus was still widely circulating in Africa as demonstrated by the identification of A/H5N1 outbreaks in other African countries, such as Ghana and Togo, between May and June 2007, and Benin in December 2007. The virus was not restricted to the poultry populations and lethal cases were also reported in humans. In the second half of March 2006, Egypt confirmed its first human case and since then, the WHO has reported 56 laboratory-confirmed human cases on the African continent of which 24 were fatal. Egypt is the African country with the highest number of human infections with 54 confirmed cases reported to date [3]. The remaining 2 human cases of HPAI infection in Africa were reported in Nigeria and in Djibouti.

At present, the availability of information on the molecular evolution of A/H5N1 in Africa is very limited. Analyses of A/H5N1 strains isolated in 2006 have shown that different sublineages were circulating in the continent, these were identified as EMA 1 and EMA2 [4] or A, B, C. [5]. As far as we are aware, information available on A/H5N1 viruses that were circulating in Africa in 2007 is limited to the results published recently on Nigerian strains isolated between January and February 2007 [6]. This study showed that the co-circulation of the 3 distinct sublineages allowed for the emergence of at least two reassortant viruses in Nigeria, one of which appears to be the predominant virus circulating in that country.

At present there is also very limited epidemiological information concerning the outbreaks in many of the affected countries and our understanding of the spread of the disease is incomplete. However, it appears to be epidemiologically complex and linked to movements of both poultry commodities and wild birds.

In the present study, we have applied molecular analysis tools to sequence data and combined the results with epidemiological data related to A/H5N1 viruses isolated between 2006 and early 2008 in all the affected African countries, namely Nigeria, Niger, Sudan, Egypt, Burkina Faso, Djibouti, Ivory Coast, Ghana, Togo, Cameroon and Benin.

## Results

A total of 494 sequences was obtained in the present study, representing the complete coding sequences for the hemagglutinin (HA) and neuraminidase (NA) gene segments of 67 strains and full sequence of the ORFs of 60 A/H5N1 HPAI viruses isolated in Africa from February 2006 to early 2008. At the time of this investigation, they represented 60% of the sequences deposited in public databases (GenBank and GISAID) originating from African viruses.

The majority of the African sequences deposited in the public databases prior to the present study were representative of the gene segments encoding the surface proteins HA and, to a lesser extent, NA. Sequences of the six internal segments generated for this study account for 74% of the total number of African internal gene segment sequences publicly available at the time of writing.

### Phylogenetic analysis

**Figure 1:**
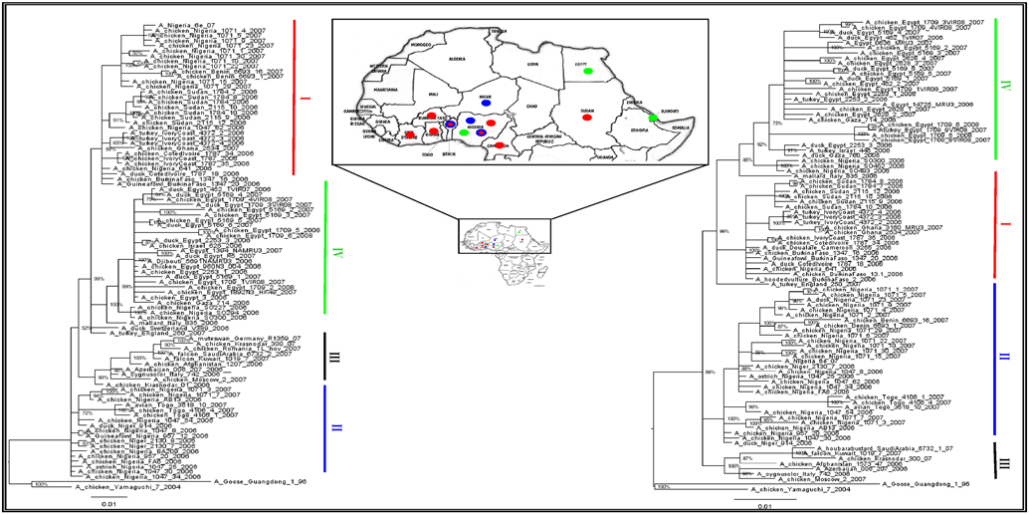
Bayesian trees for the HA (left) and NA (right) gene of A/H5N1 representative strains from Africa, Europe and Middle East (clade 2.2). Different colours are used to differentiate the viruses from distinct sublineages. Posterior probabilities are indicated above branches. Scale bar represents the number of substitutions per site. The map highlights the countries where influenza viruses were isolated. The colours of the map are the same adopted in the phylogenetic trees indicating the sublineages of clade 2.2.

Phylogenetic trees generated by Bayesian methods of the HA and NA gene segments of representative A/H5N1 viruses are presented in Figure 1. The phylogenetic tree of the HA gene generated by Bayesian methods, which includes the entire sequence dataset, as well as the trees of the 8 gene segments obtained with the neighbour-joining (N-J) method, are presented in the supplementary material (Figures S1, S2, S3, S4, S5, S6, S7, S8, S9).

Based on our phylogenetic analysis, all the African isolates analyzed in the present study are related to the A/H5N1 viruses that have been circulating in birds throughout Europe, Russia and the Middle East since late 2005. According to the unified nomenclature system for A/H5N1, they all belong to clade 2.2 [7] also described as EMA clade [4].

Detailed phylogenetic analysis of the HA gene sequences of clade 2.2 A/H5N1 viruses (figure S1) highlights the distribution of the African isolates in three distinct sublineages, here termed I, II, and IV. One additional A/H5N1 sublineage (III) was identified by phylogenetic analysis, but it does not include isolates from Africa. Representative sequences are illustrated in figure 1. With reference to the previous nomenclature [5], sublineages II, IV and I corresponded to A, B and C respectively. The grouping and tree topology remained unchanged whether using Bayesian methods or the N-J algorithm (Figure S1 and S2). The groupings were conserved using either a smaller subset of representative sequences or the entire sequence dataset (Figures 1 and S1, respectively). In particular, for the HA gene segment, grouping into I, III and IV was well supported by statistical values as indicated in figures 1 and S1. With the larger dataset (Figure S1), Bayesian posterior probabilities at the nodes were 98%; 68%; 97% and 84% for sublineages I, II, III and IV, respectively.

The remaining genes were less divergent; however, the HA gene grouping was maintained in the majority of the other genomic segments (Figures S3 to S9), namely NA, PA, PB1 and PB2. For PB1 and PB2, bootstrap values of >60 and >70 respectively were observed at the nodes.

Nucleotide similarities calculated (Kimura-2p) for 1096 nucleotide positions of 256 HA gene sequences revealed nucleotide divergence between sublineages ranging from 1.1% to 2%. The highest divergent was IV, with values ≥1.5% of nucleotide divergence from other sublineages, followed by III, with values ≥1.2%.

Higher similarity was found between sequences of viruses originating from the same country or from neighbouring countries than with other geographical areas and a noticeable association between genetic clustering and area of origin was observed in the phylogenetic trees. This finding is particularly significant for some countries as reported below.

#### Egypt

The sequences of all the Egyptian strains, isolated between 2006 and 2008 and analysed in the present study, formed a single monophyletic cluster together with sequences of the A/H5N1 viruses from Gaza and Israel. This was observed for all 8 gene segments. The Egyptian strains all clustered in the sublineage IV (figure 1). The mean nucleotide similarity for the HA gene sequences within this sublineage was 99% (Kimura-2p).

#### Western and Central Africa

During 2006–2007, all 3 sublineages (I, II and IV) were circulating in this region of Africa. A close relationship was found for strains originating from Burkina Faso, Ivory Coast, Ghana and Cameroon and their sequences formed a single cluster in sublineage I for the 8 gene segments (note: only the NA sequence is available for the strain isolated in Cameroon). Interestingly, the sequences of the viruses from Ghana, isolated in April 2007, clustered with the respective gene segments of the strains from Burkina Faso, Ivory Coast and Cameroon although these viruses were identified one year before the detection of the Ghanaian isolates. In contrast, the viruses isolated in June 2007 in Togo are genetically distinct from the isolates collected in the West African countries mentioned above, despite being isolated within a few months of each other. In the phylogenetic trees of each of the 8 gene segments, the isolates from Togo cluster with the Nigerian strains isolated in 2006 and belonging to the sublineage II. The sequence similarity between the Togolese and the Nigerian isolates for all eight gene segments ranged from 98.8% to 99.6%.

All sequences from Niger, obtained from isolates detected between February and May 2006, clustered with sequences from Nigeria isolated in 2006, and belonged to the II sublineage (sequence similarity ranging from 99.3% to 100%). Thus, they were genetically distinct from the viruses circulating in other Western and Central African countries, such as Burkina Faso, Ivory Coast, Ghana and Cameroon.

The sequences of the A/H5N1 Nigerian strains grouped in three separated sublineages I, II and IV, in agreement with an earlier publication [5].

The presence of viruses of different A/H5N1 sublineages in the same geographic region creates opportunities of reassortment. Salzberg et al [4] identified the first reassortant strain in 2006, the A/ck/Nigeria/1047-62/2006 virus. Subsequently, phylogenetic analysis performed on 12 Nigerian strains isolated at the beginning of 2007 and collected in 8 Nigerian states over a 39-day period, showed that an additional reassortant virus, with a genetic pattern distinct from that of A/ck/Nigeria/1047-62/2006 was becoming predominant and widespread in Nigeria [6]. The HA and NA genes of strain, A/Nigeria/6e/2007, isolated from a woman in the Nigerian state of Lagos, are identical to the HA and NA genes of the predominant reassortant virus detected in poultry in 2007.

In December 2007, A/H5N1 virus was reported in Benin. Full genome analysis showed that a close relationship exists between the Beninese isolates and the Nigerian strains isolated in 2007. In fact, similar reassortant A/H5N1 viruses were identified in both Western African countries.

#### Eastern Africa

The six Sudanese strains analyzed in the present study were identified in samples collected over a 15-day period in April 2006 from distinct geographical areas of the central part of the country. For each of the 8 gene segments, sequences from Sudan formed a monophyletic cluster within sublineage I. Interestingly, the Sudanese sequences were closely related to sequences from West Africa, specifically from Nigeria, Burkina Faso and Ivory Coast (e.g. a range of 99.5–99.7% sequence similarity for the HA gene), rather than to sequences from Djibouti, Egypt and the Middle East.

The sequencing of the human isolate A/Djibouti/5691/NAMRU3/06 (note: only the HA sequence is available) revealed 100% sequence similarity with another human isolate A/Egypt/2782/NAMRU3/06 and, consequently, falls into sublineage IV. (figure 1).

### Molecular characterization

For all viruses first characterized in this study, the amino acid sequences of the entire genome were determined and analysed to identify specific mutations previously identified as being associated with modified antiviral sensitivity and enhanced replication and virulence in mammalian species. In order to present a more complete picture of the genetic properties of the viruses circulating in Africa, the amino acid sequences of the African viruses available in the public database were also included in the present analysis. For ease of reference and to highlight the significance of the molecular changes observed, the molecular characterization results that appear most relevant are discussed in this section.

#### Persistent host markers in African A/H5N1 viruses

Previous studies identified host specific markers that discriminate between human influenza viruses and avian influenza viruses [8], [9]. Using multiple sequence alignments, several markers of human influenza viruses have been identified in the sequence of African A/H5N1 viruses as shown in table 1. Finkelstein et al. (2007) [8] described 13 persistent host markers, mainly distributed among PB2, PA, NP and M1 genes, which are 100% conserved in the influenza viruses that caused the 1918, 1957 and 1968 pandemics. Two of the 13 host markers in the pandemic isolates were found in the PB2 gene of African A/H5N1 isolates. In particular, all the A/H5N1 viruses isolated in this continent possess the marker E627K in the PB2 gene, which is well-known to be associated with increased virulence of A/H5N1 viruses for mice [10], [11]. The second host marker (A199S) was observed in the PB2 gene of 2 Egyptian avian viruses. This mutation was also conserved in seasonal human influenza viruses analysed in a previous study [8].

**Table 1:** Typical amino acid signature of human influenza viruses observed in the African strains.

Protein	aa position	Predicted aa		Reference	Strains	Mutation
		Avian	Human			
	661	A	T	[34]	A/ck/Ghana/2534/07	A661T
PB2				[34]		
	199	A	S	[9]	A/ck/Egypt/5169-5/07; A/dk/Egypt/5169-6/07	A199S
	627	E	K	[8]	All strains analysed in this study	E627K
	73	K	R	[9]	All strains analysed in this study belonging to IV sublineage	K73E
					A/dk/Egypt/452-1/06; A/ck/Egypt/452-2/07	
PB1-F2	82	L	S	[9]	A/dk/Egypt/5169-1/07; A/dk/Egypt/5169-4/07	L82S
					A/ck/Egypt/5169-5/07; A/Egypt/902786/06	
	79	R	Q	[9]	A/ck/Egypt/5169-3/07	R79Q
					A/ck/Nigeria/1071-3/07; A/ck/Nigeria/1071-7/07	
PA	100	V	A	[7], [34]	A/ck/Nigeria/AB13/06; A/ck/Nigeria/AB14/06	V100A
	400	Q/T/S	L	[34]	A/dk/Egypt/1709-3VIR08/07; A/ck/Egypt/2628-2/07	S400L
	356	K	R	[9]	A/ck/Burkina Faso/1347-16/06	K356R
				[34]		
NP	33	V	I	[9]	78/81 viruses	V33I
					A/ck/Egypt/5169-2/07; A/ck/Egypt/5169-3/07	
	109	I	V	[9]	A/ck/Nigeria/1047-8/06	I109V
					A/ck/Sudan/1784-8/06; A/ck/Sudan/1784-7/06	
M2	55	L	F	[34]	A/ck/Sudan/1784-10/06; A/ck/Sudan/2115-9/06	L55F
					A/ck/Sudan/2115-12/06; A/ck/Sudan/2115-10/06	
					A/hooded vulture/BurkinaFaso/2/06	
				[9]		
NS1	227	E	R o K (H1N1)	[8]	A/ck/Nigeria/FA4/06; A/ck/Nigeria/FA7/06	E227G
NS2	70	S	G	[9]	A/ck/Nigeria/FA4/06; A/ck/Nigeria/FA7/06	S70G

#### Molecular changes associated with antiviral drugs resistance

Mutations associated with modified sensitivity toward antiviral drugs have been recognized in 6 African A/H5N1 viruses.

Sequences of the M gene of 4 viruses isolated in Egypt in 2007–2008 (A/ck/Egypt/1709-5/2008, A/ck/Egypt/1709-6/2008, A/ck/Egypt/1709-8VIR08/2007 and A/tk/Egypt/1709-9VIR08/2007) showed the substitution S31N in the M2 protein (table 2). This mutation is associated with resistance to the M2 ion channel-blocking adamantane derivates and it has been reported in the M2 protein of A/H5N1 viruses isolated from humans and poultry in Hong Kong, Vietnam, Thailand, Indonesia and Cambodia [12], [13].

**Table 2:** Substitutions that can alter the sensitivity of A/H5N1 African viruses to antiviral drugs.

Susceptibility of avian influenza viruses to neuraminidase inhibitors					
Position	Residue in NA	Substitution associated with resistance	Reference	Strains	Mutation
294*	N	S	[15]	A/Egypt/14725-NAMRU3/06	N294S
				A/Egypt/14724-NAMRU3/06	

Susceptibility of avian influenza viruses to adamantanes					
Position	Residue in M2	Substitution associated with resistance	Reference	Strains	Mutation
		A or T	[33]	A/ck/Burkina Faso/13.1/06	V27I
27	V	V or A or I	[12]		
				A/avian/Togo/3618-10/07	
30	A	T or V	[33]	A/ck/Togo/4106-1/07	A30S
		S	[12]	A/ck/Togo/4106-4/07	
				A/ck/Egypt/1709-5/08	
31	S	N	[12]	A/ck/Egypt/1709-6/08	S31N
			[13]	A/ck/Egypt/1709-8VIR08/07	
				A/tk/Egypt/1709-9VIR08/07	

* N2 numbering.

Importantly, none of the mutations known to be related to resistance to neuraminidase inhibitors [14] were observed in any of the African viruses isolated from birds. However, viruses A/Egypt/14725-NAMRU3/2006 and A/Egypt/14724-NAMRU3/2006, isolated from two patients who died of pneumonia, possessed the mutation N294S (N2 numbering, table 2) [15]. The N294S substitution in the N1 subtype confers resistance to oseltamivir and a slightly reduced susceptibility to zanamivir. One A/H5N1 isolate containing this mutation had been isolated from a patient with a A/H5N1 virus infection in Vietnam after oseltamivir treatment [16]

Further molecular changes described in the literature as responsible for loss of sensitivity toward adamantane treatment have been recognised in the African isolates and are recorded in table 2.

#### Mutations in the receptor binding domains and geographical molecular signature in the HA

The receptor binding site of the HA gene of all the analysed viruses possesses amino acid residues Gln 226 and Gly 228 (H3 numbering) that preferentially recognize SAα2,3Gal linkages of avian receptors [17]. In A/H5N1 strains isolated in Egypt, several substitutions were found in the receptor binding domain, or close proximity to it (table 3). It is important to note that an A/H5N1 strain isolated in Egypt from a human case possesses the mutation S227N (H3 numbering). This mutation is located in the 220-loop of the receptor binding site (RBS) [18] and a previous study demonstrated that mutant isolates with an asparagine at position 227 may be able to influence SAα2,6Gal recognition [19]. This mutation was observed previously in A/H5N1 viruses isolated from human cases in 2003 (A/Hong Kong/212/2003 and A/Hong Kong/213/2003) [20], [21].

**Table 3:** Substitutions in the HA glycoprotein of African A/H5N1 viruses.

Virus strains	Position*1	Mutations at RBS or adjacent to it
A/Egypt/0636-NAMRU3/2007; A/Egypt/1394-NAMRU3/07; A/Egypt/2621-NAMRU3/2007	133	Deletion
A/Egypt/2629-NAMRU3/2007; A/Egypt/2631-NAMRU3/2007; A/ck/Egypt/R2/07; A/ck/Egypt/R3/07		
A/ck/Egypt/R4/07; A/ck/Egypt/R5/07; A/ck/Egypt/R6/07; A/ck/Egypt/9400NAMRU3-CLEVB211/07	155	I155T
A/chicken/Nigeria/1071-10/2007; A/chicken/Nigeria/1071-22/2007;		
A/chicken/Egypt/3044NAMRU3-CLEVB59/2007; A/chicken/Egypt/3045NAMRU3-CLEVB60/2007	133	S133L
A/chicken/Egypt/3046NAMRU3-CLEVB62/2007; A/crow/Egypt/9382NAMRU3-CLEVB111/2007		
A/chicken/Egypt/9383NAMRU3-CLEVB112/2007; A/chicken/Egypt/9384NAMRU3-CLEVB118/2007		
A/dk/Egypt/5169-1/07	186	N186S
A/Egypt/2947-NAMRU3/06	227	S227N
32 Egyptian strains	230	M230I
11 Egyptian strains	230	M230V

Virus strains	Position*2	Additional glycosylation site
A/ck/Egypt/1079-NAMRU3/07	70	NCS
A/ck/Egypt/1709-5/2008; A/ck/Egypt/1709-6/2008; A/chicken/Egypt/1709-8VIR08/2007		
A/ck/Egypt/07201-NLPQ/07; A/ck/Egypt/07202-NLPQ/07; A/ck/Egypt/07203-NLPQ/07; A/ck/Egypt/07632S-NLPQ/07; A/ck/Egypt/07701S-NLPQ/07; A/ck/Egypt/086Q-NLPQ/08; A/ck/Egypt/088S-NLPQ/08	88	NVS
A/ck/Egypt/9402NAMRU3-CLEVB213/07; A/ck/Egypt/9403NAMRU3-CLEVB214/07		
A/Egypt/6251-NAMRU3/07; A/dk/Egypt/9399NAMRU3-CLEVB202/07	252	NDT or NDS
A/chicken/Egypt/9385NAMRU3-CLEVB125/2007; A/chicken/Egypt/9386NAMRU3-CLEVB136/2007		
A/chicken/Egypt/9387NAMRU3-CLEVB148/2007; A/chicken/Egypt/9390NAMRU3-CLEVB157/2007	135	NSS
A/chicken/Egypt/9391NAMRU3-CLEVB158/2007; A/chicken/Egypt/9392NAMRU3-CLEVB167/2007		

Virus strains	Cleavage site
Isolates from Sudan	PQGEGRRKKR*GLF
Isolates from Egypt	PQGKRRRKKR*GLF
	PQRERRRKKR*GLF
	PQGEKRRKKR*GLF
	PQGERRRRKR*GLF
	PQGERRRKRR*GLF
	PQGERRRKKR*GLF
Isolates from Nigeria	PQGERRRRKR*GLF
	PQGERRRKKR*GLF
Isolates from Niger, Ivory Coast, Burkina Faso, Ghana, Benin, Togo and Djibouti	PQGERRRKKR*GLF

*1 H3 numbering.

*2 H5 numbering.

Unique amino acid signatures in the haemagglutinin molecule were identified in African viruses circulating in a given country or geographical area, specifically R341G for Sudan, P251S for Egypt, T175I for Togo, and S16G for Ivory Coast, Ghana and Burkina Faso (H5 numbering), resulting in a clear association between molecular signature and geographical origin (table 4). Statistical analysis (two-sided Fisher's exact test) confirmed that these four unique molecular signatures occurred almost exclusively within the specified countries, much more frequently than expected by chance (P<0.001, Fisher's exact test). This is also reflected in the phylogenetic analysis of the same viruses. Interestingly, the sequence derived from the human isolate from Djibouti showed the same molecular signature as the Egyptian/Palestinian/Israeli strains. It is also worth noting that the signature P251S was present in 141 out of 142 Egyptian isolates from both poultry and humans. The only exception was detected in one of the first viruses isolated in Egypt in 2005 from wild birds (A/teal/Egypt/14051-NAMRU3/2005, table 4).

**Table 4:** Unique amino acid changes in the HA protein identified in A/H5N1 viruses circulating in a given country or geographical area.

Country	aa substitution	No. HA sequences	No. HA sequences with characteristic substitution
Sudan	R341G	6	6
Egypt		142 Egypt	141 Egypt *1
Djibouti	P251S*2	1 Djibouti	1 Djibouti
Ghana		4 Ghana	4 Ghana
Ivory Coast	S16G*3	7 Ivory Coast	7 Ivory Coast
Burkina Faso		10 Burkina Faso	10 Burkina Faso
Togo	T175I	3	3

*1 except for A/teal/Egypt/14051-NAMRU3/2005.

*2 substitution observed also in the A/H5N1 strains from Gaza and Israel.

*3 substitution found also in the A/H5N1 strain A/ck/Crimea/04/2005.

## Discussion

Considering the number of sequences (n. 1152; of which 832 are from Africa) and epidemiological data collected and analysed herein, this study provides unique and comprehensive information concerning the A/H5N1 epidemic in the whole African continent from its appearance in 2006 to early 2008.

The phylogenetic results obtained in the present study indicate that, since the first Nigerian outbreak in early 2006, the Qinghai-lineage viruses (clade 2.2) has been the only clade isolated in Africa. This also sheds some light on the possible origins of the A/H5N1 viruses in Africa and confirmed previous results obtained with a smaller sequence dataset [4] indicating the viruses circulating in Russia in 2005 were the potential progenitors of the A/H5N1 virus that subsequently spread in Europe and Africa. As long as the virus continues to circulate and evolve, new clades may emerge in the future from the sublineages described here. As an example, currently circulating sublineage IV viruses in Egypt revealed genetic features that meet the criteria for a new, third order clade definition according to WHO nomenclature [7].

Shortly after the first Nigerian notification, A/H5N1 outbreaks were reported by other African countries, not only those bordering Nigeria, such as Niger, but also in more distant countries such as Burkina Faso and Egypt. The genetic analyses, performed on the A/H5N1 viruses representative of the first outbreaks in these countries, showed that 3 distinct sublineages of the A/H5N1 virus had been co-circulating since the beginning of the epidemic in Africa, suggesting multiple introductions had occurred, as reported in previous studies [4], [5], [22]. The results of the phylogenetic analysis showed that, for the period covered by this study, no further genetic sublineages were introduced into Africa. The new outbreaks subsequently reported, in chronological order, in Sudan, Ivory Coast, Djibouti, Ghana, Togo and Benin were caused by viruses all placed in the three known genetic sublineages circulating in Africa since early 2006, namely I, II and IV. In particular, it is interesting to underline that the EMA3 sublineage [4], here named sublineage III (Fig. 1), widely circulating in Europe, the Middle East and in some Asian countries during 2006 and 2007 together with the other three sublineages, had apparently not spread to Africa up to early 2008.

Some of the genetic diversity observed among viruses isolated in Africa between 2006 and early 2008 is likely to be related to the prolonged circulation of the A/H5N1 viruses in distinct poultry populations in different geographical areas, rather than to constantly recurring new virus introductions. The detailed phylogenetic and molecular analysis of A/H5N1 influenza viruses from different regions in Africa isolated between 2006 and 2007 shows that the continuing circulation of these viruses in poultry has resulted in the establishment of geographically distinct groups within the three sublineages identified in this continent. This data is confirmed by both the distinct topology of the viruses in the phylogenetic tree and the identification of unique molecular signatures in the HA sequences of the viruses circulating in a given country or region (table 4). The clustering of the African sequences with geographical origin may suggest that a certain degree of geographical segregation has occurred in Africa after the virus was first introduced into a given area. This is particularly evident for the viruses identified in Egypt, Israel and Gaza, which cluster together in the same branch of the phylogenetic tree and are distinguishable from the other African viruses. Similarly, the sequences of the isolates collected in neighbouring countries of West Africa, such as Burkina Faso, Ivory Coast and Ghana are phylogenetically closely related. It is remarkable that the Sudanese A/H5N1 viruses, which also formed a separate cluster, showed distinct genetic features compared to the sequences of the viruses isolated in the bordering or neighbouring countries, such as Egypt and Djibuti. Surprisingly, viruses circulating in Sudan were most closely related to isolates from Nigeria, Burkina Faso and Ivory Coast highlighting the complexity of the epidemiological connections within African countries and suggesting that the virus found suitable conditions to move across national borders from East to West Africa - and vice versa- by so far unidentified animate or inanimate vehicles.

Furthermore, based on the phylogenetic analyses and the genetic signatures described in the present study, the origin of the virus in Djibouti can be traced back to Egypt, Israel or Palestine. Coupling this genetic data with the epidemiological information available regarding the Djibouti case (i.e. one single non-fatal case in human, absence of reported cases of the disease in poultry, place of isolation), it seems likely that the origins of this case were in one of those three countries. From the present study, the origins and the possible roles of the location-specific amino acid signatures detected in the HA sequences (table 4) remain a matter of speculation. However, the absence of the signature P239S in one of the first viruses collected in Egypt from a wild bird, might suggest that the appearance of this distinct substitution occurred after the introduction and spread of the viruses into separate poultry populations.

Even if new genetic sublineages have not been detected in Africa in the period covered in this study, A/H5N1 viruses with a new gene constellation were identified in Nigeria in 2007 [6]. The co-circulation of viruses belonging to distinct sublineages was responsible for the emergence of a reassortant strain in Nigeria, namely EMA1/EMA2-2:6R07 virus, which corresponded to the I/II-2:6 reassortant strain according to the nomenclature used here. The present study has revealed that the spread of this reassortant was not confined to Nigeria. One A/H5N1 virus with an identical genetic pattern (I/II-2:6) was identified among samples collected during the Benin outbreak in December 2007, suggesting that this reassortant was introduced in this country from bordering Nigeria or from a common unknown.

By analysing the epidemiological and phylogenetic data presented in this study simultaneously, it was possible to determine that the initial A/H5N1 outbreaks reported in neighbouring countries Ghana and Togo, in April and June 2007 respectively, were caused by A/H5N1 viruses belonging to separate sublineages despite the geographical proximity and the narrow time period between the two outbreak notifications. These findings support the hypothesis that the Ghanaian and Togolese outbreaks are not linked epidemiologically and were the result of distinct introductions from different sources. Surprisingly, the analyses performed in the present study demonstrated that the most recent 2007 Ghanaian isolates clustered together with early strains obtained from Burkina Faso and Ivory Coast in 2006, rather than with the contemporaneous viruses identified in 2007. Considering that no further outbreaks were reported in Burkina Faso and Ivory Coast during 2007, the exact time of introduction of the A/H5N1 virus into Ghana remains obscure. It could be suggested that an early epidemiological connection between the three countries may exist or that the virus circulated undetected in that area for some time. Similarly, the origin of the 2007 Togolese strains is difficult to determine, although the phylogenetic analysis suggested that there may have been a close epidemiological link with viruses widely circulating in Nigeria in 2006.

Phylogenetic analysis of all gene segments of the A/H5N1 viruses in this study did not allow us to pinpoint the origins of the different viruses and it remains unclear how the three distinct A/H5N1 sublineages entered the continent and spread so rapidly. The difficulties encountered in tracing the spread and evolution of this virus in Africa reflect the insufficient level of resources locally available for the epidemiology, diagnosis and characterization of this infectious organism and the limitations of the surveillance programs currently implemented in some African regions.

However, based on the genetic data presented above, some hypotheses can be put forward on the introduction and mechanisms of spread of the A/H5N1 in Africa. In a similar manner to what occurred at about the same period of time in Europe, different viruses were introduced separately into Africa as already distinguishable sublineages, possibly from Central Russia [4], [23].The first detection of A/H5N1 virus in Africa occurred at a time when viruses showing common phylogeny were known to be present in Eurasian wild migratory birds [23] and such birds may have played a significant role in the introduction of the virus. After the first introduction of the A/H5N1 virus into a given area in Africa, virus populations seemed to have evolved independently, acquiring specific mutations. This suggests limited virus exchange among geographically separated susceptible host populations, which reflects the poultry economy and poultry trade in Africa. Indeed, these are mainly based on local/regional trade of rural poultry rather than on expanded/international import/export typical of the industrial poultry sectors elsewhere [24].

The application of more refined molecular tools and detailed evolutionary analysis to the genetic data now available will contribute to elucidate some aspects of this complex epidemiological situation.

In addition to virulence markers that are present in most Qinghai-lineage viruses (lineage 2.2), such as the PB2 E627K mutation, some African isolates from domestic birds exhibited mutations described as typical of human viruses in the genes encoding for internal proteins (table 1). Several mutations associated with host adaptation and to increased resistance to antiviral drugs have also been detected in the genes encoding transmembrane proteins (table 2). Although the possible epidemiological and pathogenetic roles of these amino acids substitutions have not yet been fully investigated, these findings raise concerns for the possible human health implications of viruses with these genetic characteristics.

The continued circulation of A/H5N1 viruses in the African continent not only affects the local economy but also impacts on animal and human health. It is imperative that constant efforts are undertaken to continue to monitor the evolution of A/H5N1 viruses across the African continent and to implement sustainable control strategies to improve animal and public health at a global level.

## Materials and Methods

### Samples

Between 2006 and early 2008, the OIE/FAO Reference Laboratory for avian influenza at IZSVe, Padua, Italy, received and analysed 4,587 samples collected in wild and domestic birds from 15 African countries. All the samples were screened by Real Time RT-PCR (rtRT-PCR) targeting the influenza A specific M gene [25]. Positive samples were typed by rtRT-PCR protocols specific for H5, H7 and H9 subtypes [26].

The presence of A/H5N1 virus was identified in samples collected in poultry and submitted from 9 of these countries. Sixty-seven representative African A/H5N1 strains were selected for antigenic and genomic analysis based on the country of origin, place and date of collection. Information regarding the viruses analyzed in the present study is recorded in table S1. All the laboratory analyses were performed at the IZSVe OIE/FAO Reference Laboratory. No animal experiments were conducted for this study.

### Virus isolation, nucleotide sequencing

Viruses were grown in 9- to 10-day-old embryonated specific pathogen free (SPF) fowls' eggs. The number of virus passages in eggs was limited to one in order to restrict genome modifications linked to laboratory manipulation. Subtype identification of the viruses was determined by standard haemagglutination inhibition and neuraminidase inhibition tests [27], [28].

Viral RNA was extracted from the infective allantoic fluid of SPF fowls' eggs using the Nucleospin RNA II Kit (Machery-Nagel, Duren, Germany) and was reverse transcribed with the SuperScript III Reverse Transcriptase kit (Invitrogen, Carlsbad, CA - USA). PCR amplification was performed by using specific primers (primer sequences available on request). The complete coding sequences were generated using the Big Dye Terminator v3.1 cycle sequencing kit (Applied Biosystem, Foster City, CA - USA). The products of the sequencing reactions were cleaned-up using PERFORMA DTR Ultra 96-Well kit (Edge BioSystems, Gaithersburg, MD - USA) and sequenced in a 16-capillary ABI PRISM 3130×l Genetic Analyzer (Applied Biosystem, Foster City, CA - USA). Sequences from all eight gene segments were aligned and compared with A/H5N1 sequences of viruses from Africa (*n* = 338) and with representative sequences of viruses from Europe and the Middle East (*n* = 320) available on GenBank.

### Phylogenetic analysis

Phylogenetic analyses were carried out for all the eight gene segments using the neighbour-joining (N-J) method with 1000 bootstrap replicates implemented in the MEGA 4 programme [29]. Topology of the HA and NA tree obtained was then compared to the topology of the trees generated with Bayesian methods using both the entire sequence dataset for the HA gene and a subset of sequences for the HA and NA. In detail, the selection of the most appropriate model of molecular evolution was obtained using the Akaike information criterion implemented in the computer program ModelTest v3.7 [30]. For HA and NA genes, the best-fit model of nucleotide substitution was identified as the general reversible GTR+I+Γ_4_ model, with the frequency of each substitution type, proportion of invariant sites (I), and gamma distribution of among-site rate variation with four rate categories (Γ_4_) estimated from the empirical data (parameter value available upon request). Bayesian methods implemented with the computer program MrBayes v3.1.1 [31], [32] were applied to generate the dendrograms and to assess statistical supports for the branches from 16,000 trees generated from the original dataset. The sequences obtained for this study were all deposited in public databases (GenBank and GISAID). Accession numbers are provided in Table S2.

## Supporting Information

Table S1 List of H5N1 influenza viruses sequenced and analyzed for the present study.
(0.10 MB DOC)

Table S2 List of H5N1 influenza sequences deposited for the present study
(0.09 MB DOC)

Figure S1 Bayesian trees for the HA gene of 270 H5N1 strains representative of the whole data set used in this study. Posterior probabilities are indicated at the nodes. Scale bar represents the number of substitutions per site.
(1.55 MB TIF)

Figure S2 Phylogenetic tree for the HA gene of 270 H5N1 strains representative of the whole data set used in this study. The tree was obtained using the neighbour-joining method with 1000 bootstrap replicates implemented in the MEGA 4 programme. Bootstrap values >50 are indicated at the nodes.
(8.25 MB TIF)

Figure S3 Phylogenetic tree for the NA gene of 148 H5N1 strains representative of the whole data set used in this study. The tree was obtained using the neighbour-joining method with 1000 bootstrap replicates implemented in the MEGA 4 programme. Bootstrap values >50 are indicated at the nodes.
(3.74 MB TIF)

Figure S4 Phylogenetic tree for the M gene of 126 H5N1 strains representative of the whole data set used in this study. The tree was obtained using the neighbour-joining method with 1000 bootstrap replicates implemented in the MEGA 4 programme. Bootstrap values >50 are indicated at the nodes.
(5.44 MB TIF)

Figure S5 Phylogenetic tree for the PB1 gene of 134 H5N1 strains representative of the whole data set in this study. The tree was obtained using the neighbour-joining method with 1000 bootstrap replicates implemented in the MEGA 4 programme. Bootstrap values >50 are indicated at the nodes.
(4.90 MB TIF)

Figure S6 Phylogenetic tree for the PB2 gene of 120 H5N1 strains representative of the whole data set used in this study. The tree was obtained using the neighbour-joining method with 1000 bootstrap replicates implemented in the MEGA 4 programme. Bootstrap values >50 are indicated at the nodes.
(3.90 MB TIF)

Figure S7 Phylogenetic tree for the PA gene of 121 H5N1 strains representative of the whole data set in this study. The tree was obtained using the neighbour-joining method with 1000 bootstrap replicates implemented in the MEGA 4 programme. Bootstrap values >50 are indicated at the nodes.
(3.52 MB TIF)

Figure S8 Phylogenetic tree for the NP gene of 117 H5N1 strains representative of the whole data set used in this study. The tree was obtained using the neighbour-joining method with 1000 bootstrap replicates implemented in the MEGA 4 programme. Bootstrap values >50 are indicated at the nodes.
(4.20 MB TIF)

Figure S9 Phylogenetic tree for the NS gene of 123 H5N1 strains representative of the whole data set used in this study. The tree was obtained using the neighbour-joining method with 1000 bootstrap replicates implemented in the MEGA 4 programme. Bootstrap values >50 are indicated at the nodes.
(3.90 MB TIF)
