# Supplementary material for: Highly Pathogenic Avian Influenza Virus Subtype H5N1 in Africa: A Comprehensive Phylogenetic Analysis and Molecular Characterization of Isolates
Source: PLoS One. 2009 Mar 17;4(3):e4842. doi: 10.1371/journal.pone.0004842 (PMC2653644; doi:10.1371/journal.pone.0004842)
Supplement: Table S1 — List of H5N1 influenza viruses sequenced and analyzed for the present study. (0.10 MB DOC) [file pone.0004842.s001.doc]

**Table S1**: List of H5N1 influenza viruses sequenced and analyzed for the present study.

| Viruses | **Data** | **Location** |
| --- | --- | --- |
| Influenza A virus (A/chicken/Egypt/2253-1/2006(H5N1)) | 12.02.06 | Giza |
| Influenza A virus (A/turkey/Egypt/2253-2/2006(H5N1)) | 12.02.06 | Menia |
| Influenza A virus (A/duck/Egypt/2253-3/2006(H5N1)) | 02.05.06 | Dakhalia |
| Influenza A virus (A/duck/Egypt/452-1/2006(H5N1)) | 22.11.06 | Gharbia |
| Influenza A virus (A/chicken/Egypt/452-2/2007(H5N1)) | 02.01.07 | Gharbia |
| Influenza A virus (A/chicken/Egypt/2628-1/2007(H5N1)) | 22.02.07 | Fayom |
| Influenza A virus (A/chicken/Egypt/2628-2/2007(H5N1)) | 11.03.07 | Damietta |
| Influenza A virus (A/chicken/Egypt/2628-3/2007(H5N1)) | 11.03.07 | Damietta |
| Influenza A virus (A/chicken/Egypt/2628-4/2007(H5N1)) | 05.04.07 | Dakahlia |
| Influenza A virus (A/duck/Egypt/5169-1/2007(H5N1)) | 11.04.2007 | Damitta |
| Influenza A virus (A/chicken/Egypt/5169-2/2007(H5N1)) | 04.06.2007 | Menia |
| Influenza A virus (A/chicken/Egypt/5169-3/2007(H5N1)) | 15.6.2007 | Assuit |
| Influenza A virus (A/duck/Egypt/5169-4/2007(H5N1)) | 17.02.2007 | Gharbia |
| Influenza A virus (A/chicken/Egypt/5169-5/2007(H5N1)) | 20.06.2007 | Luxor |
| Influenza A virus (A/duck/Egypt/5169-6/2007(H5N1)) | 20.06.2007 | Luxor |
| Influenza A virus (A/chicken/Egypt/1709-1VIR08/2007(H5N1)) | 25.02.2007 | Dakahlia |
| Influenza A virus (A/chicken/Egypt/1709-2/2008(H5N1)) | 05.01.2008 | Gharbia |
| Influenza A virus (A/duck/Egypt/1709-3VIR08/2007(H5N1)) | 04.03.2007 | Fayom |
| Influenza A virus (A/chicken/Egypt/1709-4VIR08/2007(H5N1)) | 04.03.2007 | Alexandria |
| Influenza A virus (A/chicken/Egypt/1709-5/2008(H5N1)) | 08.01.2008 | Sharkia |
| Influenza A virus (A/chicken/Egypt/1709-6/2008(H5N1)) | 03.01.2008 | Quena |
| Influenza A virus (A/chicken/Egypt/1709-8VIR08/2007(H5N1)) | 18.12.2007 | Kaliobia |
| Influenza A virus (A/turkey/Egypt/1709-9VIR08/2007(H5N1)) | 20.12.2007 | Sharkia |
| Influenza A virus (A/chicken/Sudan/1784-7/2006(H5N1) | 25.04.06 | Soba |
| Influenza A virus (A/chicken/Sudan/1784-8/2006(H5N1) | 25.04.06 | Soba |
| Influenza A virus (A/chicken/Sudan/1784-10/2006(H5N1) | 25.04.06 | Butri |
| Influenza A virus (A/chicken/Sudan/2115-9/2006(H5N1)) | 15.04.06 | Kuku |
| Influenza A virus (A/chicken/Sudan/2115-10/2006(H5N1)) | 15.04.06 | Tayba Alhasanab |
| Influenza A virus (A/chicken/Sudan/2115-12/2006(H5N1)) | 16.04.06 | Alhaj Yousuf |
| Influenza A virus (A/turkey/Ivory Coast/4372-2/2006(H5N1)) | 9.11.06 | Abidjan |
| Influenza A virus (A/turkey/Ivory Coast/4372-3/2006(H5N1)) | 9.11.06 | Abidjan |
| Influenza A virus (A/turkey/Ivory Coast/4372-4/2006(H5N1)) | 9.11.06 | Abidjan |
| Influenza A virus (A/duck/Ivory Coast/1787-18/2006(H5N1)) | 13.03.06 | Abidjan |
| Influenza A virus (A/chicken/Ivory Coast/1787-34/06(H5N1) | 06.04.06 | Abidjan |
| Influenza A virus (A/chicken/Ivory Coast/1787-35/06(H5N1) | 06.04.06 | Abidjan |
| Influenza A virus (A/chicken/Ghana/2534/2007(H5N1)) | 24.04.07 | Tema |
| Influenza A virus (A/chicken/Togo/3618-10/2007(H5N1)) | 14.06.07 | Aneho |
| Influenza A virus (A/ chicken /Togo/4106-1/07(H5N1)) | 16.06.07 | Agbata |
| Influenza A virus (A/ chicken /Togo/4106-4/07(H5N1)) | 16.06.07 | Adetikope |
| Influenza A virus (A/chicken/Nigeria/641/2006(H5N1) | 17.01.06 | Kaduna State |
| Influenza A virus (A/guinea fowl/Nigeria/957-12/2006(H5N1)) | 23.02.06 | Bauchi |
| Influenza A virus (A/chicken/Nigeria/957-20/2006(H5N1)) | 23.02.06 | Jos |
| Influenza A virus (A/duck/Niger/914/2006(H5N1)) | 14.02.06 | Magaria |
| Influenza A virus (A/chicken/Niger/2130-7/2006(H5N1)) | 11.05.06 | Mai Gao |
| Influenza A virus (A/chicken/Niger/2130-8/2006(H5N1)) | 11.05.06 | Mai Gao |
| Influenza A virus (A/chicken/Burkina Faso/1347-16/2006(H5N1)) | 23.03.06 | Saaba |
| Influenza A virus (A/guinea fowl /Burkina Faso/1347-20/2006(H5N1)) | 23.03.06 | Saaba |
| Influenza A virus (A/chicken/Nigeria/1047-8/2006(H5N1)) | 01.03.06 | Bauchi |
| Influenza A virus (A/ostrich/Nigeria/1047-25/2006(H5N1)) | 23.03.06 | Bauchi |
| Influenza A virus (A/chicken/Nigeria/1047-54/2006(H5N1)) | 06.06.06 | Oyo |
| Influenza A virus (A/chicken/Nigeria/1047-62/2006(H5N1)) | 07.06.06 | Taraba |
| Influenza A virus (A/chicken/Nigeria/1047-30/2006(H5N1)) | 22.02.06 | Beneu |
| Influenza A virus (A/chicken/Nigeria/1047-34/2006(H5N1)) | 25.02.06 | Abuja (FCT) |
| Influenza A virus (A/chicken/Nigeria/1071-1/2007(H5N1)) | 02.01.07 | Plateau |
| Influenza A virus (A/chicken/Nigeria/1071-3/2007(H5N1)) | 05.01.07 | Sokoto |
| Influenza A virus (A/chicken/Nigeria/1071-4/2007(H5N1)) | 05.01.07 | Borno |
| Influenza A virus (A/chicken/Nigeria/1071-5/2007(H5N1)) | 06.01.07 | Plateau |
| Influenza A virus (A/chicken/Nigeria/1071-7/2007(H5N1)) | 10.01.07 | Sokoto |
| Influenza A virus (A/chicken/Nigeria/1071-9/2007(H5N1)) | 12.01.07 | Bauchi |
| Influenza A virus (A/chicken/Nigeria/1071-10/2007(H5N1)) | 13.01.07 | Anambra |
| Influenza A virus (A/chicken/Nigeria/1071-15/2007(H5N1)) | 23.01.07 | Kaduna |
| Influenza A virus (A/chicken/Nigeria/1071-22/2007 (H5N1)) | 31.01.07 | Kano |
| Influenza A virus (A/chicken/Nigeria/1071-23/2007(H5N1)) | 01.02.07 | Borno |
| Influenza A virus (A/chicken/Nigeria/1071-29/2007(H5N1)) | 09.02.07 | Lagos |
| Influenza A virus (A/chicken/Nigeria/1071-30/2007(H5N1)) | 10.02.07 | Kaduna |
| Influenza A virus (A/chicken/Benin/6693-16/2007(H5N1)) | 04/12/07 | Cotonou |
| Influenza A virus (A/chicken/Benin/6693-1/2007(H5N1)) | 04/12/07 | Honvié |
