# Supplementary material for: Highly Pathogenic Avian Influenza Virus Subtype H5N1 in Africa: A Comprehensive Phylogenetic Analysis and Molecular Characterization of Isolates
Source: PLoS One. 2009 Mar 17;4(3):e4842. doi: 10.1371/journal.pone.0004842 (PMC2653644; doi:10.1371/journal.pone.0004842)
Supplement: Table S2 — List of H5N1 influenza sequences deposited for the present study (0.09 MB DOC) [file pone.0004842.s002.doc]

**Table S2**: List of H5N1 influenza sequences deposited for the present study

| Viruses | **Accession number** |
| --- | --- |
| Influenza A virus (A/chicken/Egypt/2253-1/2006(H5N1)) | CY020645 to CY020652 |
| Influenza A virus (A/turkey/Egypt/2253-2/2006(H5N1)) | CY020653 to CY020660 |
| Influenza A virus (A/duck/Egypt/2253-3/2006(H5N1)) | CY016899 to CY016906 |
| Influenza A virus (A/duck/Egypt/452-1/2006(H5N1)) | EPI156730 to EPI156737 |
| Influenza A virus (A/chicken/Egypt/452-2/2007(H5N1)) | EU373735, EPI154900 to EPI154906 |
| Influenza A virus (A/chicken/Egypt/2628-1/2007(H5N1)) | EU373736; EPI154951 to EPI154957 |
| Influenza A virus (A/chicken/Egypt/2628-2/2007(H5N1)) | EPI156745; EPI156746 |
| Influenza A virus (A/chicken/Egypt/2628-3/2007(H5N1)) | EPI156747; EPI156748 |
| Influenza A virus (A/chicken/Egypt/2628-4/2007(H5N1)) | EPI156749; EPI156750 |
| Influenza A virus (A/duck/Egypt/5169-1/2007(H5N1)) | EU373737; EPI152036 to EPI152042 |
| Influenza A virus (A/chicken/Egypt/5169-2/2007(H5N1)) | EPI152043 to EPI152050 |
| Influenza A virus (A/chicken/Egypt/5169-3/2007(H5N1)) | EPI156767 to EPI156774 |
| Influenza A virus (A/duck/Egypt/5169-4/2007(H5N1)) | EPI156775 to EPI156782 |
| Influenza A virus (A/chicken/Egypt/5169-5/2007(H5N1)) | EPI156783, EPI156784 |
| Influenza A virus (A/duck/Egypt/5169-6/2007(H5N1)) | EPI156785, EPI156786 |
| Influenza A virus (A/chicken/Egypt/1709-1VIR08/2007(H5N1)) | EU717849; EU717850  EPI154907 to EPI154912 |
| Influenza A virus (A/chicken/Egypt/1709-2/2008(H5N1)) | EPI154913 to EPI154920 |
| Influenza A virus (A/duck/Egypt/1709-3VIR08/2007(H5N1)) | EU717851; EU717852  EPI154921 to EPI154926 |
| Influenza A virus (A/chicken/Egypt/1709-4VIR08/2007(H5N1)) | EU717853; EU717854  EPI154927 to EPI154932 |
| Influenza A virus (A/chicken/Egypt/1709-5/2008(H5N1)) | EU17855, EU17856; EPI154933 to EPI154938 |
| Influenza A virus (A/chicken/Egypt/1709-6/2008(H5N1)) | EU17857, EU17858; EPI154939 to EPI154943 |
| Influenza A virus (A/chicken/Egypt/1709-8VIR08/2007(H5N1)) | EPI159882 to EPI159889 |
| Influenza A virus (A/turkey/Egypt/1709-9VIR08/2007(H5N1)) | EPI154944 to EPI154950 |
| Influenza A virus (A/chicken/Sudan/1784-7/2006(H5N1) | CY016292 to CY016299 |
| Influenza A virus (A/chicken/Sudan/1784-8/2006(H5N1) | CY020661 to CY020668 |
| Influenza A virus (A/chicken/Sudan/1784-10/2006(H5N1) | CY016300 to CY016307 |
| Influenza A virus (A/chicken/Sudan/2115-9/2006(H5N1)) | CY020669 to CY020676 |
| Influenza A virus (A/chicken/Sudan/2115-10/2006(H5N1)) | CY21389 to CY21396 |
| Influenza A virus (A/chicken/Sudan/2115-12/2006(H5N1)) | CY20677 to CY20684 |
| Influenza A virus (A/turkey/Ivory Coast/4372-2/2006(H5N1)) | CY020693 to CY020700 |
| Influenza A virus (A/turkey/Ivory Coast/4372-3/2006(H5N1)) | CY020701 to CY020708 |
| Influenza A virus (A/turkey/Ivory Coast/4372-4/2006(H5N1)) | CY020709 to CY020716 |
| Influenza A virus (A/duck/Ivory Coast/1787-18/2006(H5N1)) | CY016803 to CY016810 |
| Influenza A virus (A/chicken/Ivory Coast/1787-34/06(H5N1) | CY016811 to CY016818 |
| Influenza A virus (A/chicken/Ivory Coast/1787-35/06(H5N1) | CY021517 to CY021524 |
| Influenza A virus (A/chicken/Ghana/2534/2007(H5N1)) | EU373734; EPI151933 to EPI151936; EPI151941 to EPI151943 |
| Influenza A virus (A/avian/Togo/3618-10/2007(H5N1)) | EPI156751 to EPI156758 |
| Influenza A virus (A/ chicken /Togo/4106-1/07(H5N1)) | EU373738; EPI151944 to EPI151949; EPI151956 |
| Influenza A virus (A/ chicken /Togo/4106-4/07(H5N1)) | EPI156759 to EPI156766 |
| Influenza A virus (A/chicken/Nigeria/641/2006(H5N1) | CY016276 to CY016283 |
| Influenza A virus (A/guinea fowl/Nigeria/957-12/2006(H5N1)) | CY017179 to CY017186 |
| Influenza A virus (A/chicken/Nigeria/957-20/2006(H5N1)) | CY016284 to CY016291 |
| Influenza A virus (A/duck/Niger/914/2006(H5N1)) | CY017027 to CY017034 |
| Influenza A virus (A/chicken/Niger/2130-7/2006(H5N1)) | DQ838517; EPI156738 to EPI156744 |
| Influenza A virus (A/chicken/Niger/2130-8/2006(H5N1)) | DQ838516 |
| Influenza A virus (A/chicken/Burkina Faso/1347-16/2006(H5N1)) | EU277833 to EU277840 |
| Influenza A virus (A/guinea fowl /Burkina Faso/1347-20/2006(H5N1)) | EU277841 to EU277848 |
| Influenza A virus (A/chicken/Nigeria/1047-8/2006(H5N1)) | CY016907 to CY016914 |
| Influenza A virus (A/ostrich/Nigeria/1047-25/2006(H5N1)) | CY016915 to CY016922 |
| Influenza A virus (A/chicken/Nigeria/1047-54/2006(H5N1)) | CY016923 to CY016930 |
| Influenza A virus (A/chicken/Nigeria/1047-62/2006(H5N1)) | CY016931 to CY016938 |
| Influenza A virus (A/chicken/Nigeria/1047-30/2006(H5N1)) | CY016939 to CY016946 |
| Influenza A virus (A/chicken/Nigeria/1047-34/2006(H5N1)) | CY016947 to CY016954 |
| Influenza A virus (A/chicken/Nigeria/1071-1/2007(H5N1)) | EU148356 to EU148363 |
| Influenza A virus (A/chicken/Nigeria/1071-3/2007(H5N1)) | EU148364 to EU148371 |
| Influenza A virus (A/chicken/Nigeria/1071-4/2007(H5N1)) | EU148372 to EU148379 |
| Influenza A virus (A/chicken/Nigeria/1071-5/2007(H5N1)) | EU148380 to EU148387 |
| Influenza A virus (A/chicken/Nigeria/1071-7/2007(H5N1)) | EU148388 to EU148395 |
| Influenza A virus (A/chicken/Nigeria/1071-9/2007(H5N1)) | EU148396 to EU148403 |
| Influenza A virus (A/chicken/Nigeria/1071-10/2007(H5N1)) | EU148404 to EU148411 |
| Influenza A virus (A/chicken/Nigeria/1071-15/2007(H5N1)) | EU148412 to EU148419 |
| Influenza A virus (A/chicken/Nigeria/1071-22/2007 (H5N1)) | EU148420 to EU148427 |
| Influenza A virus (A/chicken/Nigeria/1071-23/2007(H5N1)) | EU148428 to EU148435 |
| Influenza A virus (A/chicken/Nigeria/1071-29/2007(H5N1)) | EU148436 to EU148443 |
| Influenza A virus (A/chicken/Nigeria/1071-30/2007(H5N1)) | EU148444 to EU148451 |
| Influenza A virus (A/chicken/Benin/6693-16/2007(H5N1)) | EU436612; EPI152005 to EPI152011 |
| Influenza A virus (A/chicken/Benin/6693-1/2007(H5N1)) | EPI156787; EPI156788 |
