# Supplementary figures and images for: Highly Pathogenic Avian Influenza Virus Subtype H5N1 in Africa: A Comprehensive Phylogenetic Analysis and Molecular Characterization of Isolates
Source: PLoS One. 2009 Mar 17;4(3):e4842. doi: 10.1371/journal.pone.0004842 (PMC2653644; doi:10.1371/journal.pone.0004842)

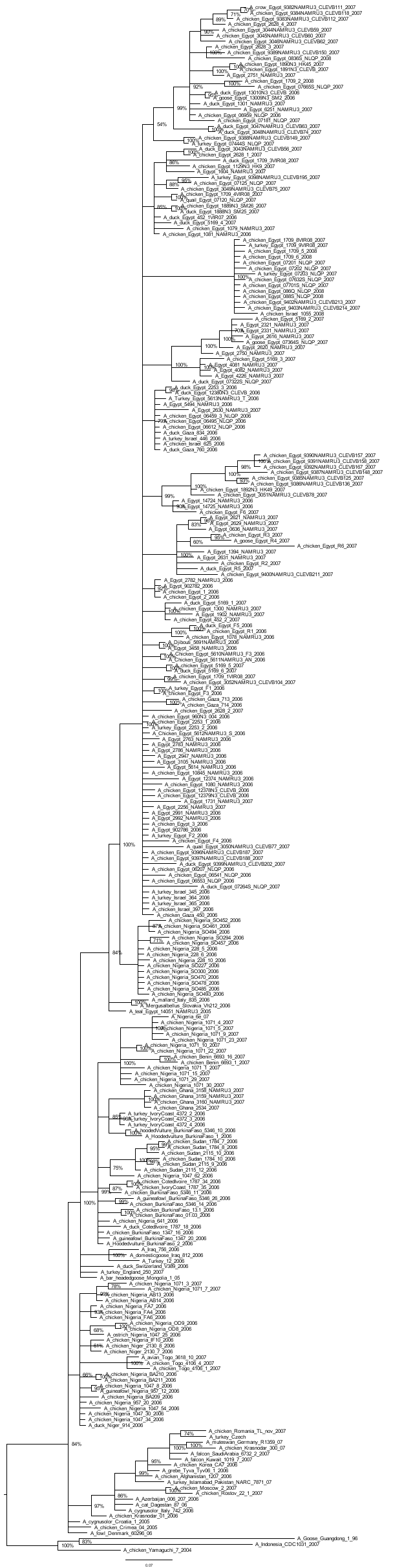

Supplement: Figure S1 — Bayesian trees for the HA gene of 270 H5N1 strains representative of the whole data set used in this study. Posterior probabilities are indicated at the nodes. Scale bar represents the number of substitutions per site. (1.55 MB TIF) [file pone.0004842.s003.tif]

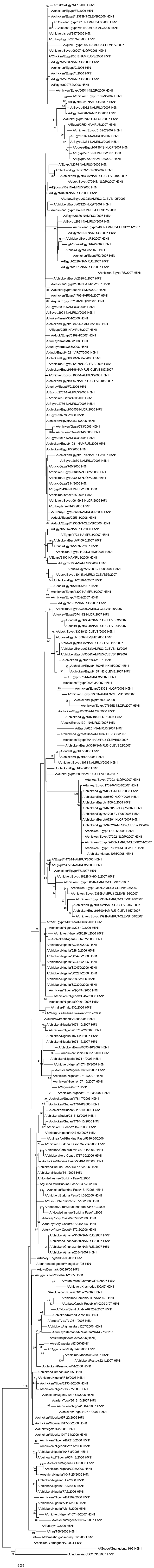

Supplement: Figure S2 — Phylogenetic tree for the HA gene of 270 H5N1 strains representative of the whole data set used in this study. The tree was obtained using the neighbour-joining method with 1000 bootstrap replicates implemented in the MEGA 4 programme. Bootstrap values >50 are indicated at the nodes. (8.25 MB TIF) [file pone.0004842.s004.tif]

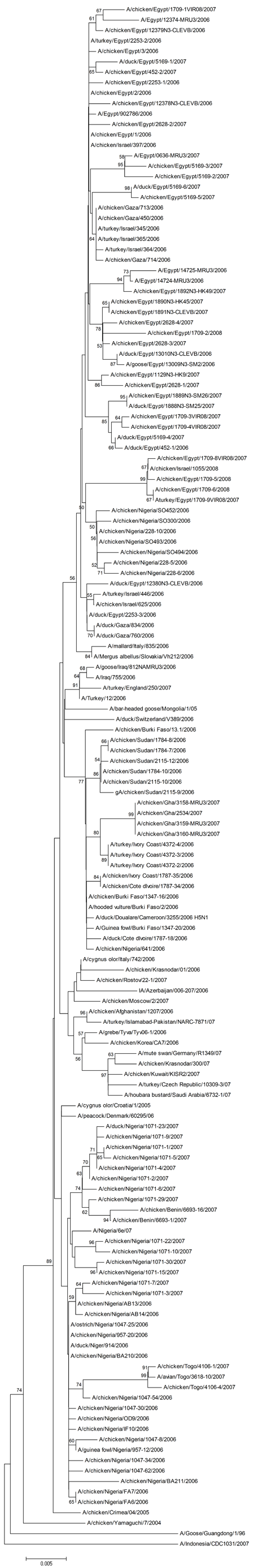

Supplement: Figure S3 — Phylogenetic tree for the NA gene of 148 H5N1 strains representative of the whole data set used in this study. The tree was obtained using the neighbour-joining method with 1000 bootstrap replicates implemented in the MEGA 4 programme. Bootstrap values >50 are indicated at the nodes. (3.74 MB TIF) [file pone.0004842.s005.tif]

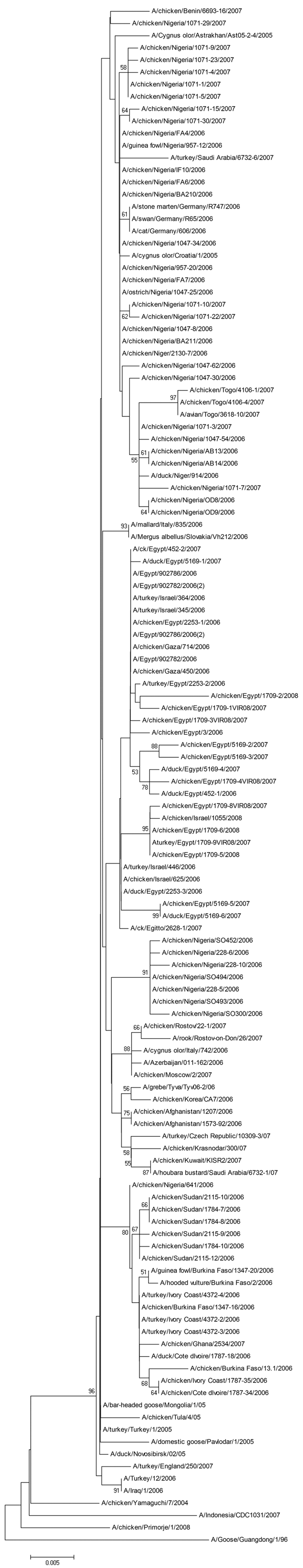

Supplement: Figure S4 — Phylogenetic tree for the M gene of 126 H5N1 strains representative of the whole data set used in this study. The tree was obtained using the neighbour-joining method with 1000 bootstrap replicates implemented in the MEGA 4 programme. Bootstrap values >50 are indicated at the nodes. (5.44 MB TIF) [file pone.0004842.s006.tif]

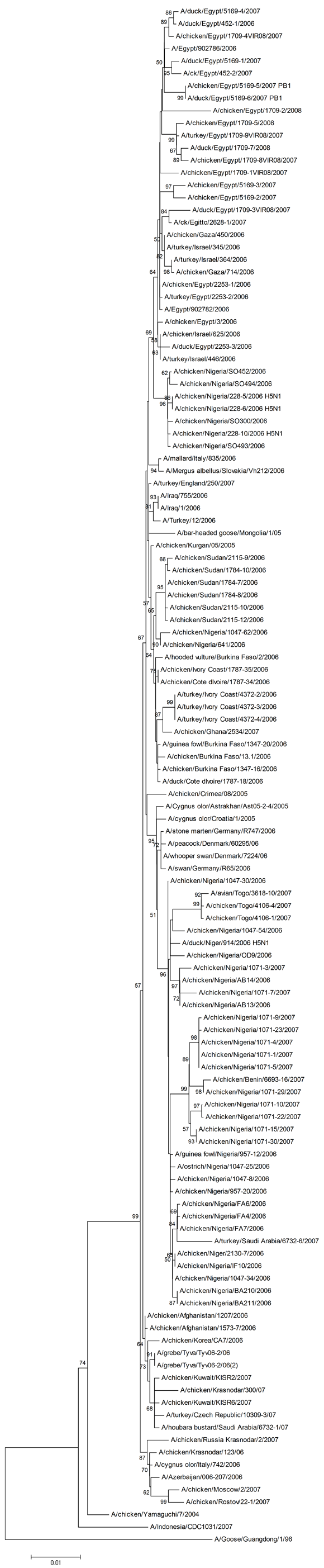

Supplement: Figure S5 — Phylogenetic tree for the PB1 gene of 134 H5N1 strains representative of the whole data set in this study. The tree was obtained using the neighbour-joining method with 1000 bootstrap replicates implemented in the MEGA 4 programme. Bootstrap values >50 are indicated at the nodes. (4.90 MB TIF) [file pone.0004842.s007.tif]

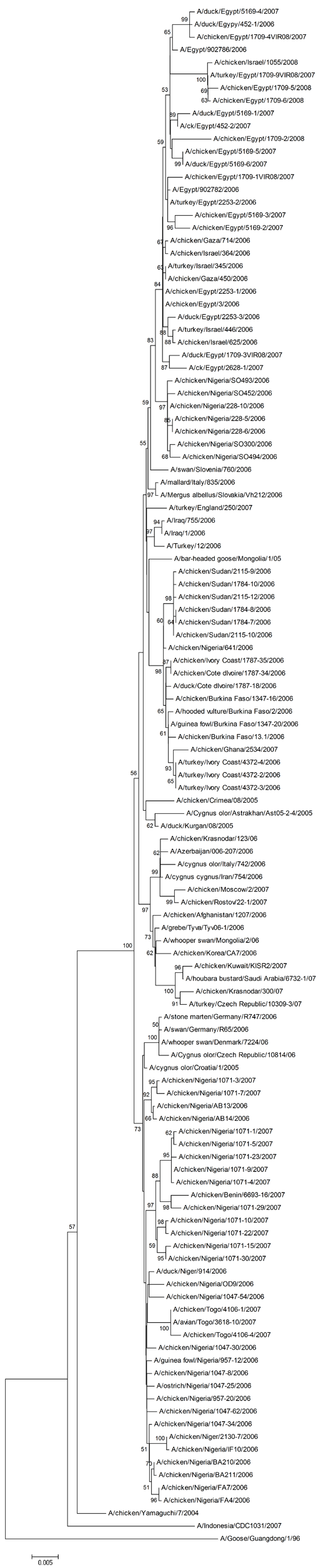

Supplement: Figure S6 — Phylogenetic tree for the PB2 gene of 120 H5N1 strains representative of the whole data set used in this study. The tree was obtained using the neighbour-joining method with 1000 bootstrap replicates implemented in the MEGA 4 programme. Bootstrap values >50 are indicated at the nodes. (3.90 MB TIF) [file pone.0004842.s008.tif]

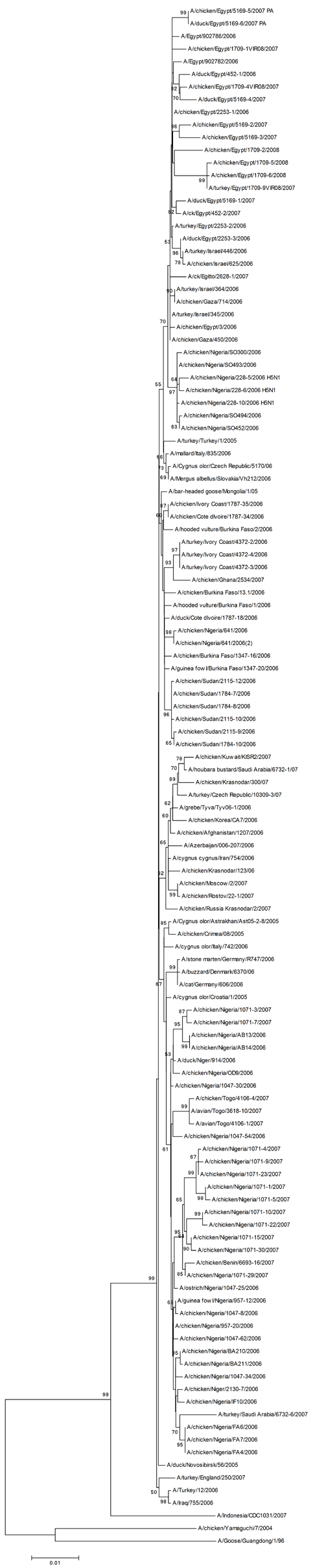

Supplement: Figure S7 — Phylogenetic tree for the PA gene of 121 H5N1 strains representative of the whole data set in this study. The tree was obtained using the neighbour-joining method with 1000 bootstrap replicates implemented in the MEGA 4 programme. Bootstrap values >50 are indicated at the nodes. (3.52 MB TIF) [file pone.0004842.s009.tif]

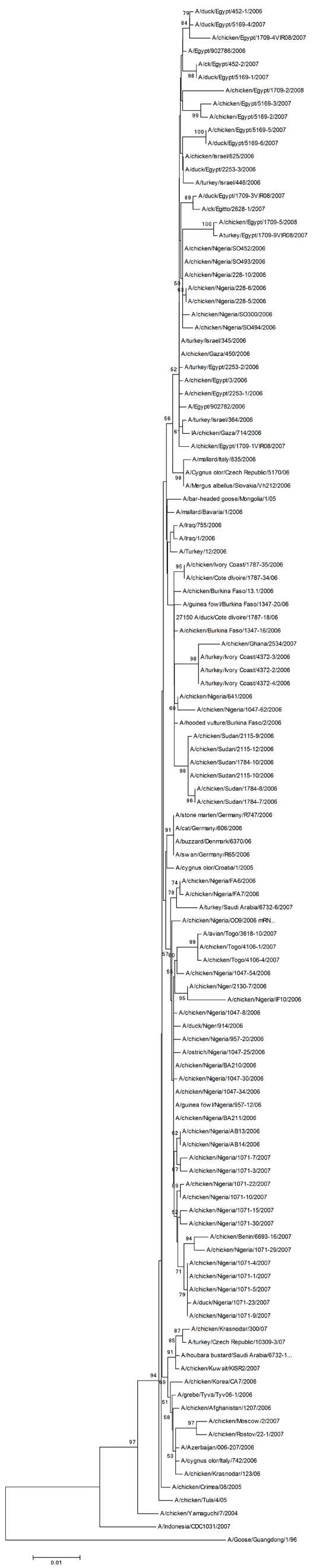

Supplement: Figure S8 — Phylogenetic tree for the NP gene of 117 H5N1 strains representative of the whole data set used in this study. The tree was obtained using the neighbour-joining method with 1000 bootstrap replicates implemented in the MEGA 4 programme. Bootstrap values >50 are indicated at the nodes. (4.20 MB TIF) [file pone.0004842.s010.tif]

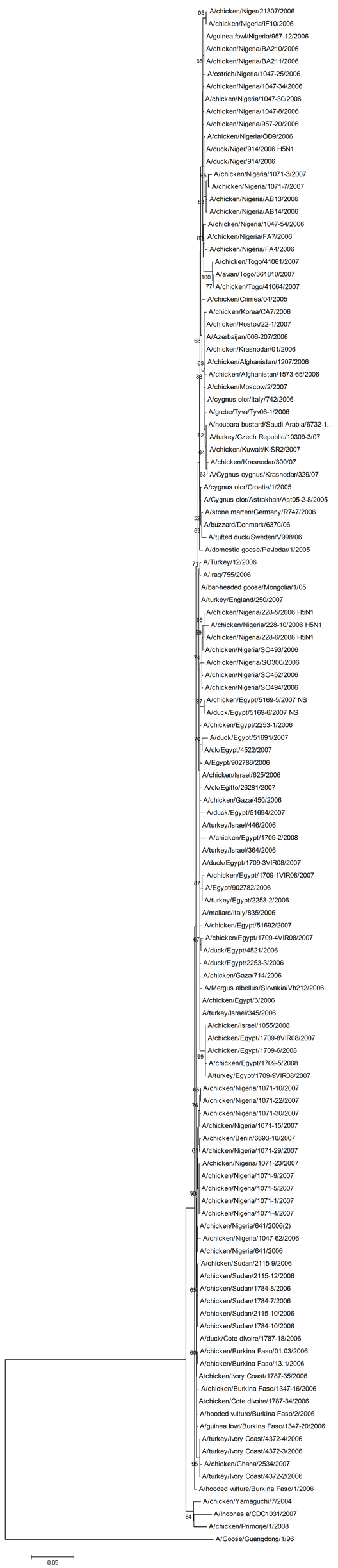

Supplement: Figure S9 — Phylogenetic tree for the NS gene of 123 H5N1 strains representative of the whole data set used in this study. The tree was obtained using the neighbour-joining method with 1000 bootstrap replicates implemented in the MEGA 4 programme. Bootstrap values >50 are indicated at the nodes. (3.90 MB TIF) [file pone.0004842.s011.tif]
